# How Thermal Modification Affects the Short-Term Bending Creep Behaviour of Fraxinus Excelsior Wood

**DOI:** 10.3390/ma19183892

**Published:** 2026-09-12

**Authors:** Przemysław Mania, Mateusz Fertsch, Magdalena Broda

**Affiliations:** Department of Wood Science and Thermal Techniques, Faculty of Forestry and Wood Technology, Poznan University of Life Sciences, ul. Wojska Polskiego 38/42, 60-637 Poznan, Poland; mateuszfer96@gmail.com

**Keywords:** time-dependent behaviour, ash wood, heat treatment, creep compliance, bending creep

## Abstract

**Highlights:**

**What are the main findings?**

• Thermally modified ash wood exhibits lower absolute creep compliance (*I*_24_) than untreated wood across all evaluated load levels.
• Anti-creep efficiency (ACE_24_) and absolute compliance reduction increase with increasing stress level (40–60% MOR).

**What are the implications of the main findings?**

• Thermal treatment can reduce the tendency of ash wood to creep under short-term loading.

**Abstract:**

Among wood types used in industry, ash wood is one of the most commonly heat-treated species. It is widely applied for outdoor applications such as cladding, terrace boards, and garden furniture, as well as in indoor applications that require high dimensional stability. Although the mechanical properties of thermally modified ash have been extensively studied, its creep behaviour under sustained load remains insufficiently described. This study evaluates the short-term creep behaviour of ash wood thermally modified at 210 °C under bending stress, based on deflection measurements at different stress levels and under controlled humidity conditions. Untreated ash wood with comparable moisture content served as a reference. The results show that thermally modified wood exhibited lower creep compliance than untreated wood at all applied stress levels. After 24 h of loading, the absolute reduction in total creep compliance was 3.7% at 40% stress and 20.3% at 60% stress relative to control wood. The difference between modified and unmodified wood became more pronounced as the load level increased. These findings indicate that thermal modification can reduce the tendency of ash wood to creep under short-term loading conditions. Given the limited test duration and the use of small specimens, the results should be interpreted with caution and cannot be directly extrapolated to structural-scale applications. Nevertheless, the study provides new knowledge and is an interesting basis for further investigation of the creep behaviour of thermally modified wood under long-term loading and variable environmental conditions.

## 1. Introduction

Thermal modification is the most advanced, environmentally friendly, and economically significant commercial wood modification method, enabling the production of timber with reduced hygroscopicity, improved dimensional stability, and enhanced durability. The process usually occurs at temperatures between 160 and 240 °C under either “wet” or “dry” conditions, depending on the wood species and the desired properties of the final product [1,2,3,4]. Thermal modification of wood involves a series of chemical transformations, primarily including the degradation of hemicelluloses, deacetylation, and structural changes in lignin. Although oxidative reactions may occur, particularly in the presence of oxygen during the early stages of the process, their contribution is limited under typical industrial conditions, where reduced-oxygen environments are used [2,5,6]. Although the extent of chemical changes is moderate, it translates into reduced wood hygroscopicity, which contributes to enhanced decay resistance and dimensional stability compared to unmodified timber. The process also darkens wood colour and improves acoustic properties [7,8,9,10,11,12]. Therefore, thermal modification is an excellent method for enhancing the selected properties of low-durability local wood, making it suitable for outdoor applications or purposes that require high dimensional stability, including decking, exterior cladding and joinery [1,13,14,15,16].

Unfortunately, alterations in the chemical composition and structure of the main cell wall polymers reduce wood density, thereby affecting the mechanical properties of thermally treated wood. Although the process enhances the surface hardness of timber, it considerably deteriorates other mechanical properties, including stiffness, as well as shear, compression and bending strength, thereby limiting its applications for structural purposes [9,12,17,18,19,20]. It should be noted, however, that from the perspective of structural applications, creep in wood also plays an essential role in the mechanical stability and safety of the construction [21,22].

Creep is defined as the time-dependent deformation of timber subjected to a constant load (or stress) over a long period. It results from the viscoelastic nature of wood, in which the viscous component allows slow deformation over time, even when the applied stress is below the wood’s immediate strength. Creep is not just a theoretical issue—it is a design-critical factor for all wood structures under continuous load, and ignoring it can lead to severe serviceability and structural integrity problems, as well as costly repairs [23,24].

The research on creep behaviour of timber revealed that it is affected by the moisture content and temperature (higher moisture and temperatures accelerate creep), load level (higher sustained loads result in higher creep), as well as the wood species and density (higher density usually translates to reduced creep) [22,23,25]. It has been shown that thermal modification affects the creep behaviour of wood through fundamental structural changes in the wood cell wall components [26]. Researchers also analysed creep in wood under variable climate conditions [27], creep behaviour of compression wood [28], investigated the effects of various modifications on the wood creep [29,30,31,32,33,34,35] and studied the bending and compressive creep behaviour of sawn lumber beams for four years to provide an approximate formula for predicting creep behaviour of construction wood in the long-term [36].

Although the wood creep behaviour is relatively well recognised, there are still gaps in knowledge of creep in thermally modified timber, as only a few scientific reports address this topic. For example, Hoseinzadeh et al. examined heat-treated beech wood and found that the macroscopic creep modulus declines when treatment temperatures exceed 160 °C, due to bulk thermal degradation and mass loss [26]. However, studies at the micro- and nanoscale—using nanoindentation to assess the cell wall show that high-temperature treatments (160–220 °C) markedly decrease the intrinsic creep rate of individual wood cell walls [37,38,39]. This apparent contradiction across scales can be explained by the difference between bulk structure and cell-wall polymer chemistry: while high-temperature modification lowers overall density and static elastic modulus due to hemicellulose degradation, it also reduces hygroscopicity, increases cellulose crystallinity, and promotes thermal cross-linking of lignin. These changes restrict molecular chain slippage, thereby strengthening the cell-wall polymer network against time-dependent creep [2,26,37,40]. The results of research by Xu et al. on Japanese cedar wood subjected to hydrothermal treatment at 180 °C for four hours indicate that the applied treatment significantly enhances the wood’s creep resistance [41]. In turn, thermo-hydro-mechanical treatment of beech (*Fagus sylvatica* L.) wood resulted in decreased creep and recovery compliances as compared to control specimens [42]. Majka and Roszyk studied the creep of heat-treated ash wood subjected to compression in tangential and radial directions, and simultaneously wetted from 6% moisture content to above the fibre saturation point, revealed that thermal treatment reduces the strain of wood subjected to compression perpendicular to the grain to a degree proportional to the mass loss [43]. Research on wood creep behaviour at the nano scale (cell wall level) revealed that high temperature heat treatments (160–220 °C) reduce the creep rate of wood cell walls, which results from changes in the chemistry of wood polymers: degradation of hemicelluloses, increase in cellulose crystallinity, recondensation of lignin, cellulose microfibrils rearrangement and cross-linking between cell wall polymers [37,38,40]. Therefore, our studies aimed to improve the understanding of the creep behaviour of thermally modified wood.

As a research material, ash wood (*Fraxinus excelsior* L.) was selected. European ash is among the most commonly heat-treated wood species. The modification significantly reduces wood hygroscopicity, enhancing its dimensional stability, surface quality, as well as resistance to fungal decay and weathering factors [44,45,46,47,48,49], thus making it a popular raw material for outdoor use, such as claddings, facades, terrace boards, garden furniture, as well as for many indoor applications requiring high dimensional accuracy, including panelling boards, floorboards, and stairs [3,50].

The multiple applications of heat-treated ash have led to extensive studies on how treatment conditions affect its mechanical behaviour, as thermal modification also influences wood’s mechanical properties. Researchers investigated the fracture behaviour and compression strength of ash modified at different temperatures, showing that its brittleness increases and compression strength decreases with increasing treatment temperature [9,51,52,53]. Standfest and Zimmer [54] demonstrated that thermal treatment at 160, 180, and 200 °C enhances the ash Brinell hardness in a longitudinal direction. However, the highest applied temperature reduces hardness in both radial and tangential directions, whereas the two lower temperatures do not affect these properties. In contrast, Govorčin et al. [55] showed hardness reduction in all anatomical directions for ash treated at 200 °C. Hannouz et al. and Govorčin et al. [19,55] found that heat treatment at temperatures of 200–210 °C reduces the strength properties of ash wood except for compression parallel to the grain, and increases its modulus of elasticity perpendicular to the grain. Roszyk et al. [50] reported that heat treatment at 180 °C and 200 °C reduced the hygroscopicity of ash wood, resulting in enhanced strength and elasticity measured in compression for wet wood compared to untreated and treated samples. Multivariate analysis revealed that the modification temperature had a significant impact on compression strength parallel to the grain, whereas the strongest effect on the modulus of elasticity was observed perpendicular to the grain.

Despite the research on the mechanical properties of thermally modified ash conducted so far, its behaviour under load, particularly creep, has not yet been investigated. Therefore, our study focused on a better understanding of the creep behaviour of thermally modified ash wood by conducting experiments under bending stress. The goal of the experiments was to determine the creep curves of heat-treated ash wood from deflection measurements under various bending stresses and constant humidity conditions. For comparison, the experiment was also performed on unmodified ash wood samples to verify the effect of thermal modification on creep. The research results demonstrate that thermal modification significantly enhances short-term viscoelastic creep resistance, providing valuable insights into the time-dependent mechanical stability of modified timber.

## 2. Materials and Methods

### 2.1. Materials

The research material comprised clear, straight-grained European ash wood (*Fraxinus excelsior* L.) obtained from commercial sources in the Wielkopolska region, Poland. Part of the wood was thermally modified at 210 °C in the sawmill Tartak Stefan (Włoszakowice, Poland) using the ThermoWood method [56] under a superheated steam atmosphere. The treatment schedule comprised three stages (heating/drying, thermal modification, and cooling/conditioning), with the peak temperature maintained at 210 ± 2 °C for 3 h, resulting in a mean oven-dry mass loss of 9.2 ± 0.6%. Unmodified wood served as a control (CTR). All specimens were visually pre-screened to ensure complete freedom from structural defects, knots, reaction wood, and grain deviations, with growth rings oriented strictly parallel to the faces of the specimens. For measurements, wood samples with dimensions of 5 mm × 10 mm × 150 mm (in the tangential, radial, and longitudinal directions, respectively) were prepared. For the flexural creep evaluation, a total of 108 specimens were initially tested at three load levels (40%, 50%, and 60% of MOR). Two specimens from the CTR 40% group exhibiting localised structural defects and abnormally high deflections were excluded. The final analysed dataset comprised N = 106 specimens (*n* = 16 for CTR-40%, and *n* = 18 per load level for all remaining groups).

### 2.2. Methods

#### 2.2.1. Determination of the Average Bending Strength of Unmodified and Thermally Modified Wood

The determination of bending strength was preceded by the controlled selection of specimens to minimise material variability. After preparation, the density of all samples was determined using a stereometric method. The resulting density distribution was analysed, and specimens with densities near the median value (50th percentile) were selected for further testing. This procedure ensured a homogeneous sample set and reduced the confounding influence of density on the evaluation of mechanical properties and creep behaviour.

The density of each sample was determined using a stereometric method in accordance with ISO 13061-2 (ISO 13061-2, 2014) [57], based on oven-dry mass and oven-dry volume, after the specimens were dried to constant mass. Wood dimensions and mass were measured using a digital calliper with an accuracy of 0.01 mm and an analytical balance with an accuracy of 0.001 g (Sartorius GmbH, Göttingen, Germany), respectively. Then, the bending strength (modulus of rupture, MOR) was determined in a three-point bending test using a numerically controlled Zwick ZO50TH (Zwick/Roell, Ulm, Germany) universal testing machine. The specimens had nominal dimensions of 10 × 10 × 150 mm, and the span length was set to 120 mm. Loading was applied in the tangential direction. To ensure anatomical equivalence, static bending and creep specimens were prepared as adjacent twin samples from the same wood strips. The specimens are termed ‘twin samples’ because each 5 × 10 × 150 mm creep specimen was cut directly adjacent to a corresponding 10 × 10 × 150 mm static (MOR) specimen within the same wooden strip. This ensured that paired specimens shared identical annual growth rings, grain orientation, and anatomical characteristics. Static reference MOR was determined on 10 × 10 × 150 mm specimens (L/h = 12, 120 mm span). Because creep specimens were tested at h = 5 mm (L/h = 24, 120 mm span), stressed-volume size effects yield a higher true MOR for the shallower geometry (estimated +7–9% for control wood according to Weibull volumetric size-effect models [58] with m = 8–10). Importantly, thermal modification increases wood brittleness [3], resulting in a lower Weibull modulus (m) for TM wood and a larger size-effect correction. Consequently, the true operational stress ratio for TM specimens is lower than the nominal values, thereby slightly inflating the apparent advantage of the modification relative to control wood.

The obtained values of bending strength and elastic modulus for thermally modified (TM) and control unmodified samples (CTR) are presented in Table 1.

Thermal treatment at 210 °C resulted in mass loss and degradation of cell walls, as is typical of high-temperature processes. This caused the average oven-dry density to drop from 609 ± 11.5 kg m^−3^ (CTR) to 545 ± 19.5 kg m^−3^ (TM), a decrease of about 10.5% (*p* < 0.001). At the same time, the mean bending strength (MOR) fell from 144 ± 16.3 MPa to 127 ± 28.4 MPa (11.8%). In contrast, the Modulus of Elasticity (MOE) showed a slight increase from 13.5 ± 1.51 GPa to 14.9 ± 2.75 GPa.

The moisture content of thermally modified ash wood immediately after modification was about 1.6%, while the control wood had a moisture content of approximately 6%. Because this study aimed to determine creep curves at similar moisture levels, all samples underwent a conditioning process. Thermal modification fundamentally reduces the hygroscopicity of wood cell walls by degrading the hydroxyl groups (-OH) in hydrophilic amorphous hemicellulose [2]. Consequently, conditioning both material variants under identical environmental conditions would result in differing internal moisture contents, thereby conflating intrinsic cell wall modifications with moisture-induced plasticisation effects. To isolate the intrinsic viscoelastic response of the polymer, the equilibrium moisture content (EMC) was standardised across variants to approximately 10%, corresponding with the typical equilibrium moisture content of wood used in indoor residential applications. To compensate for the reduced hygroscopicity of the thermally modified wood and achieve a target equilibrium moisture content comparable to the control samples (EMC ≈ 9.1–9.4%), modified specimens were conditioned at a higher relative humidity. Specifically, control samples were seasoned under laboratory conditions at 20 ± 2 °C and 55 ± 2% relative humidity (RH) for 6 weeks. The thermally modified samples were conditioned for the same period in desiccators over a saturated NaCl salt solution at 20 ± 1 °C (RH = 75%). Following conditioning, the moisture content (MC) was determined stereometrically, yielding average baseline values of 9.4% for control samples and 9.1% for thermally modified samples.

#### 2.2.2. Measurement of Mid-Span Deflection

Following the baseline static bending tests (Section 2.2.1), loads corresponding to 40%, 50%, and 60% of the average ultimate flexural strength (MOR) of each material variant were calculated to evaluate mid-span creep deflection. For unmodified samples, the applied loads were 8 kg, 10 kg, and 12 kg; for thermally modified samples, they were 7 kg, 8.8 kg, and 10.6 kg, respectively.

**Figure 1:**
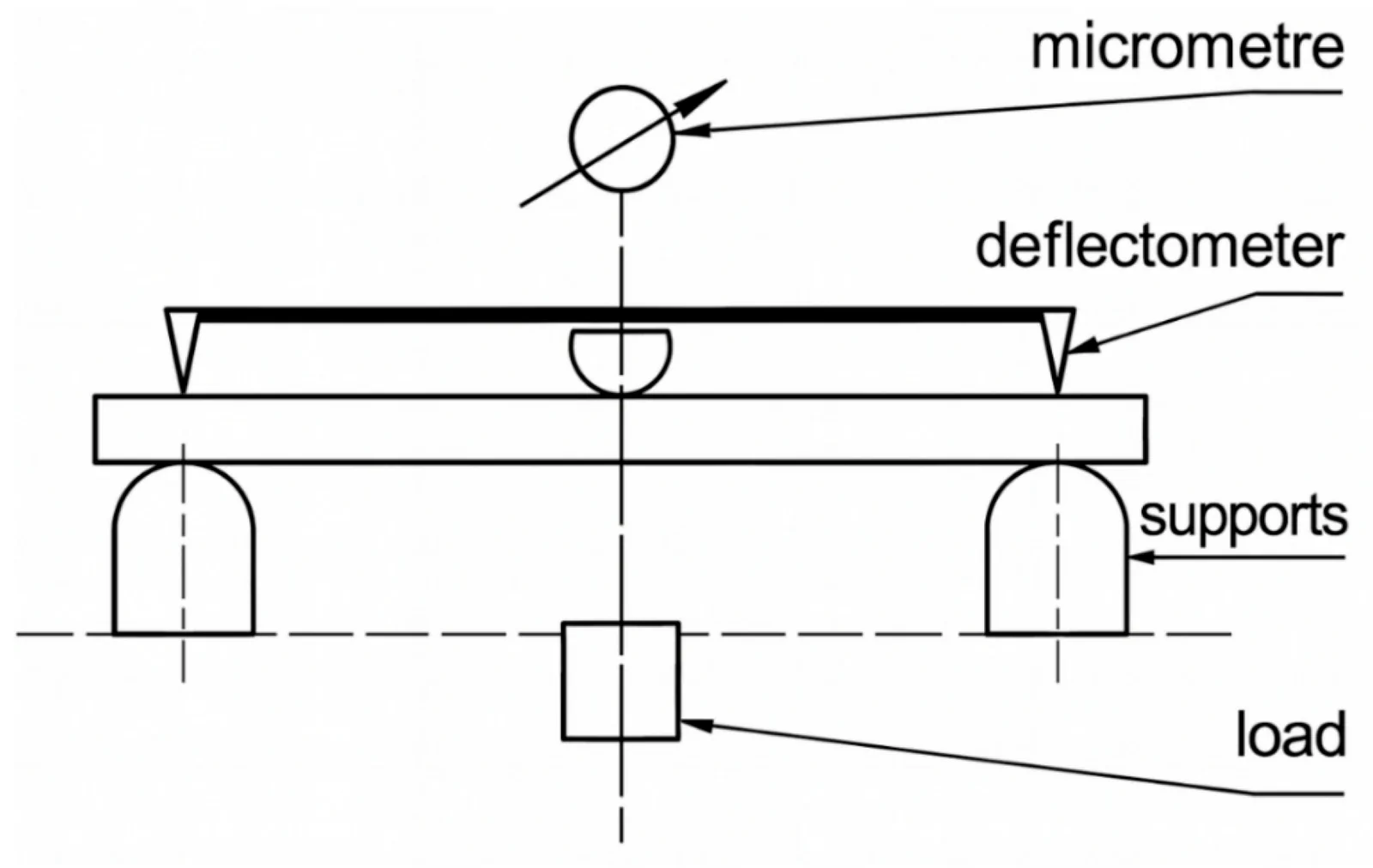
Diagram of the prototype equipment for testing creep in wood under bending stress used in the study.

A sample was placed on a prototype creep-testing apparatus (Figure 1) so that the force was applied exactly at the midpoint of its length, and the force acted parallel to the tangents of the annual growth rings. The spacing between the supports (L) was 120 mm. Next, the deflectometer was set up so that its blades were in contact with the sample at its support points, while the micrometre’s measuring pin rested on the flat surface of the load. Mid-span vertical deflection was monitored using a digital micrometre with a resolution of 0.001 mm. The sensor pin rested on the central loading platen, which was equipped with a cylindrical contact nose (R = 10 mm) to distribute the applied stress and minimise localised transverse compression. Considering the high stiffness of the steel load frame and the small contact stresses, transverse indentation and apparatus compliance were estimated to contribute less than 0.8% to the total measured deflection, exerting an identical systematic effect across all experimental groups. After zeroing the micrometre, the load was applied to a sample, and the immediate deflection (δ) was read. Subsequent measurements were read every minute for the first 5 min, then every 5 min from 5 to 30 min, every half hour from 0.5 to 4 h, and every hour from 4 to 6 h. The last measurement was taken 24 h after the test began. Further measurements were considered pointless due to the small secondary increments.

Viscoelastic creep testing for CTR specimens was conducted in a climate-controlled enclosure (20 ± 0.5 °C, 55 ± 2% RH), while TM specimens were tested in a climatic chamber (20 ± 0.5 °C, 75 ± 2% RH) to ensure target EMC normalisation (≈9.1–9.4%). Temperature and relative humidity were logged continuously throughout each 24-h test. Specimen mass was recorded immediately before and after testing, showing negligible weight change (Δm < 0.1%), confirming bulk moisture equilibrium throughout the testing period. While micro-gradients cannot be completely ruled out, continuous monitoring confirmed stable ambient conditions (20 ± 0.5 °C; RH = 55 ± 2% for CTR, 75 ± 2% for TM) throughout all 24 h runs, thereby minimising transient moisture fluctuations that drive mechano-sorptive creep.

Creep compliance (*I_t_*) for each load applied was calculated based on Equation (1):

(1) $$I_{t}=\frac{\varepsilon_{t}}{\sigma },[{\mathrm{MPa}}^{-1}]$$

where *ε* is strain $\varepsilon =\frac{6h\delta }{L^{2}}$ (mm\mm), and *σ* is stress $\sigma =\frac{3FL}{2bh^{2}}$ (MPa).

Relative creep compliance (*Z*) was calculated according to Equation (2):

(2) $$Z=\frac{I_{t}}{I_{0}}$$

where *I_t_* is creep compliance at the moment *t* of the measurement, and *I*_0_ is instantaneous creep compliance.

The anti-creep efficiency (*ACE*) measures the treatment’s effectiveness in reducing creep deformation relative to untreated wood, with positive values indicating improvement and negative values indicating reduced efficiency. It was calculated based on Equation (3):

(3) $${ACE}_{t}=\frac{I_{u}-I_{t}}{I_{u}}\cdot 100$$

where *I_u_*—creep compliance of untreated (control) wood, *I_t_*—creep compliance of thermally modified wood.

To quantitatively compare long-term creep kinetics, time-dependent compliance *I*(*t*) was fitted to Findley’s power-law model:

(4) *I*(*t*) = *I*_0_ + *a*∙*t*^*n*^

where *I*_0_ is the instantaneous compliance, *a* is the creep amplitude coefficient, and *n* is the dimensionless time exponent. Model parameters were determined using non-linear least-squares regression.

#### 2.2.3. Statistical Analysis

Statistical analyses were performed with Statistica 13.3 (TIBCO Software Inc., Palo Alto, CA, USA). Normality and homogeneity of variance were assessed using the Shapiro–Wilk test (*p* > 0.05) and Levene’s test (*p* > 0.05), respectively. Homoscedasticity was verified using Levene’s test of equality of variance (W(5100) = 1.84, *p* = 0.112), confirming that the assumption of equal variances was satisfied across treatment groups prior to two-way ANOVA. To evaluate the main and interaction effects of treatment type and load intensity, a two-way ANOVA was conducted on instantaneous compliance (*I*_0_), total compliance at 24 h (*I*_24_), and relative compliance over 24 h (*Z*_24_), considering two fixed factors: Material Variant (2 levels: CTR, TM) and Stress Level (3 levels: 40%, 50%, 60% MOR). When significant interactions between Material and Stress Level were detected, post hoc pairwise comparisons were conducted using Tukey’s HSD test at α = 0.05. A one-way ANOVA was used to examine differences in *ACE* over time across stress levels at 24 h. All results are expressed as mean ± standard deviation (SD).

## 3. Results and Discussion

**Figure 2:**
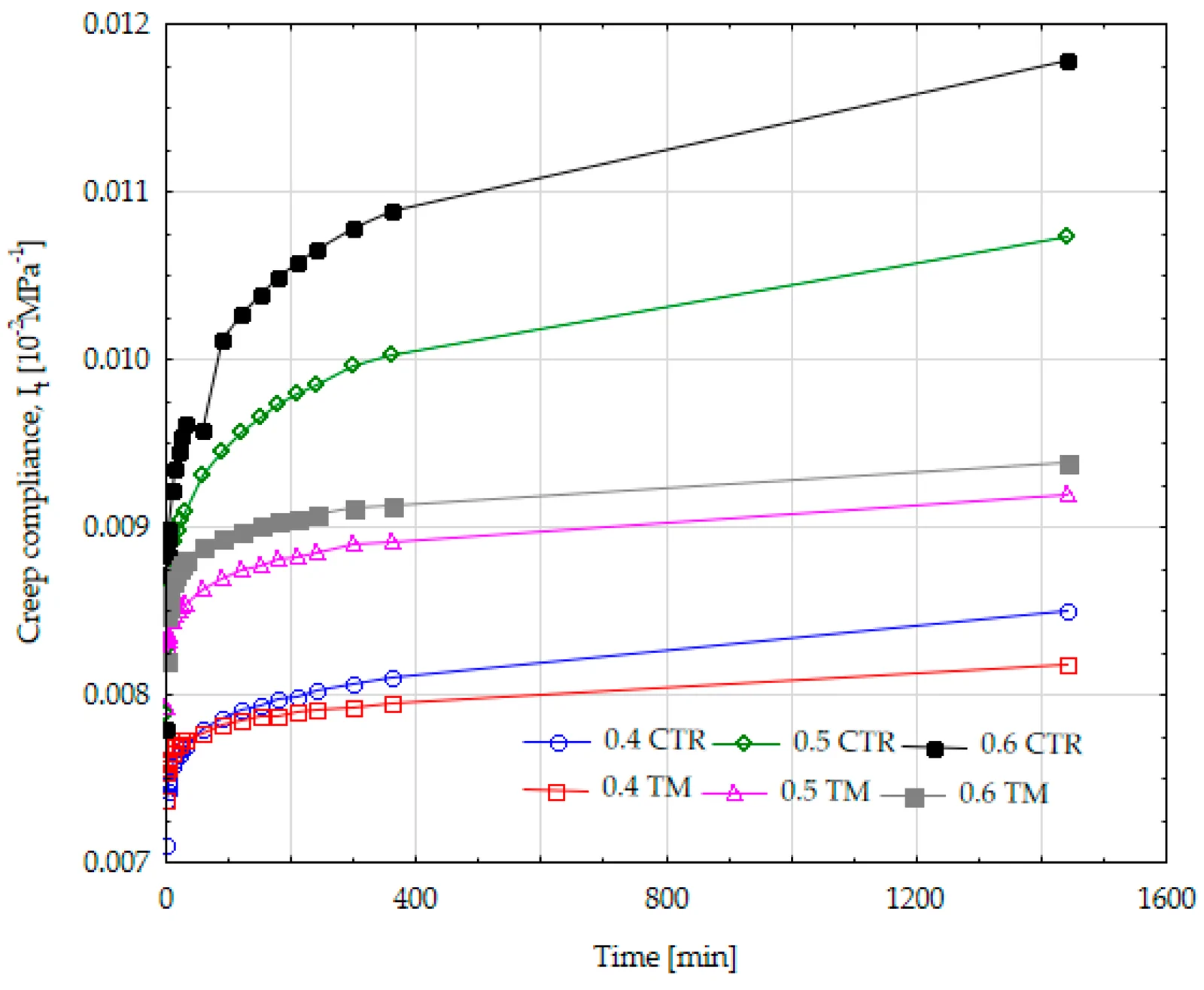
Average creep compliance *I_t_* (expressed in 10^−2^ MPa^−1^) of unmodified (CTR) and thermally modified (TM) ash wood under bending stresses corresponding to 40% (0.4), 50% (0.5), and 60% (0.6) of the maximum bending strength of wood.

**Figure 3:**
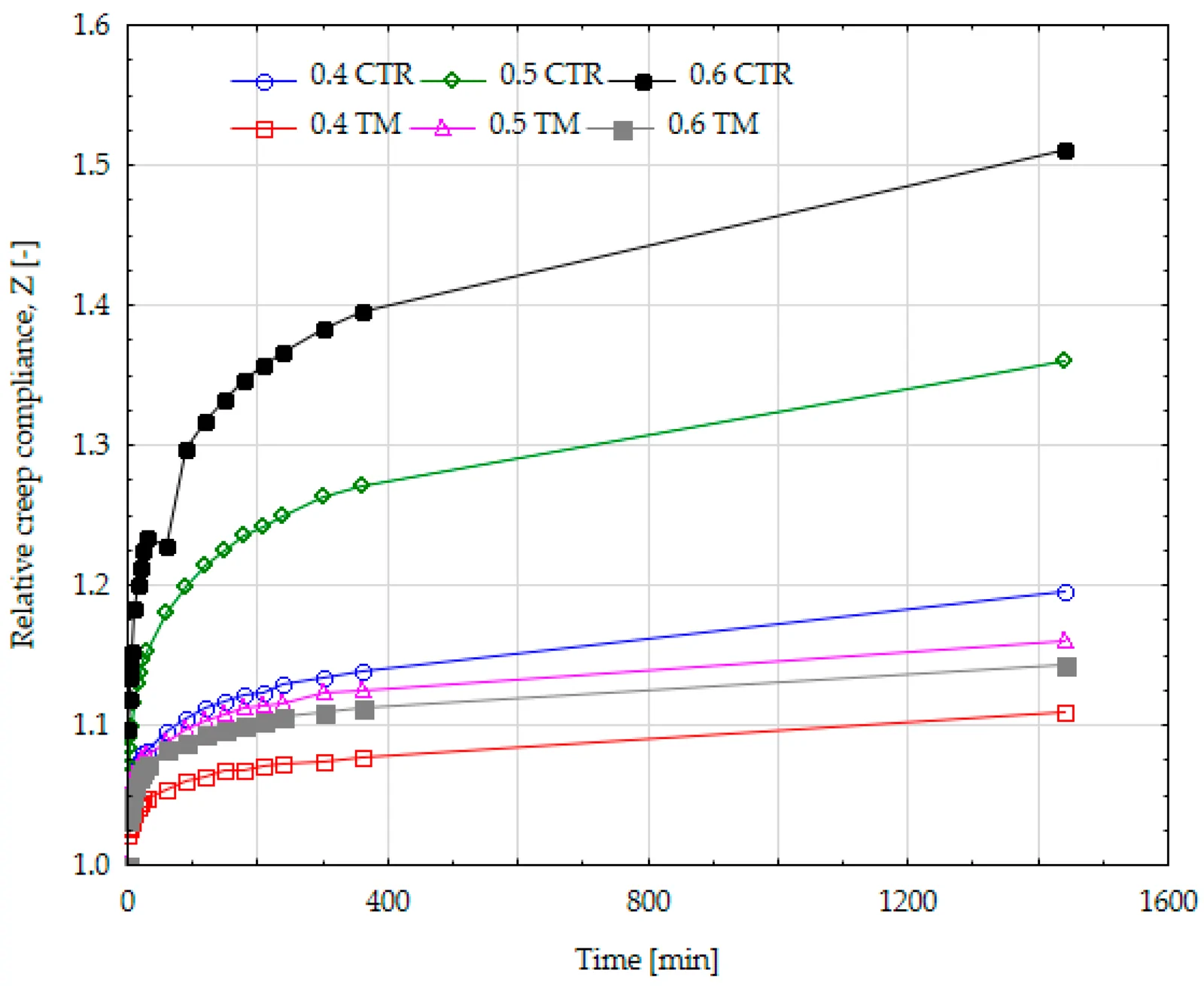
Average relative creep compliance *Z* (dimensionless) of unmodified (CTR) and thermally modified (TM) ash wood under bending stresses corresponding to 40% (0.4), 50% (0.5), and 60% (0.6) of the maximum bending strength of wood.

**Figure 4:**
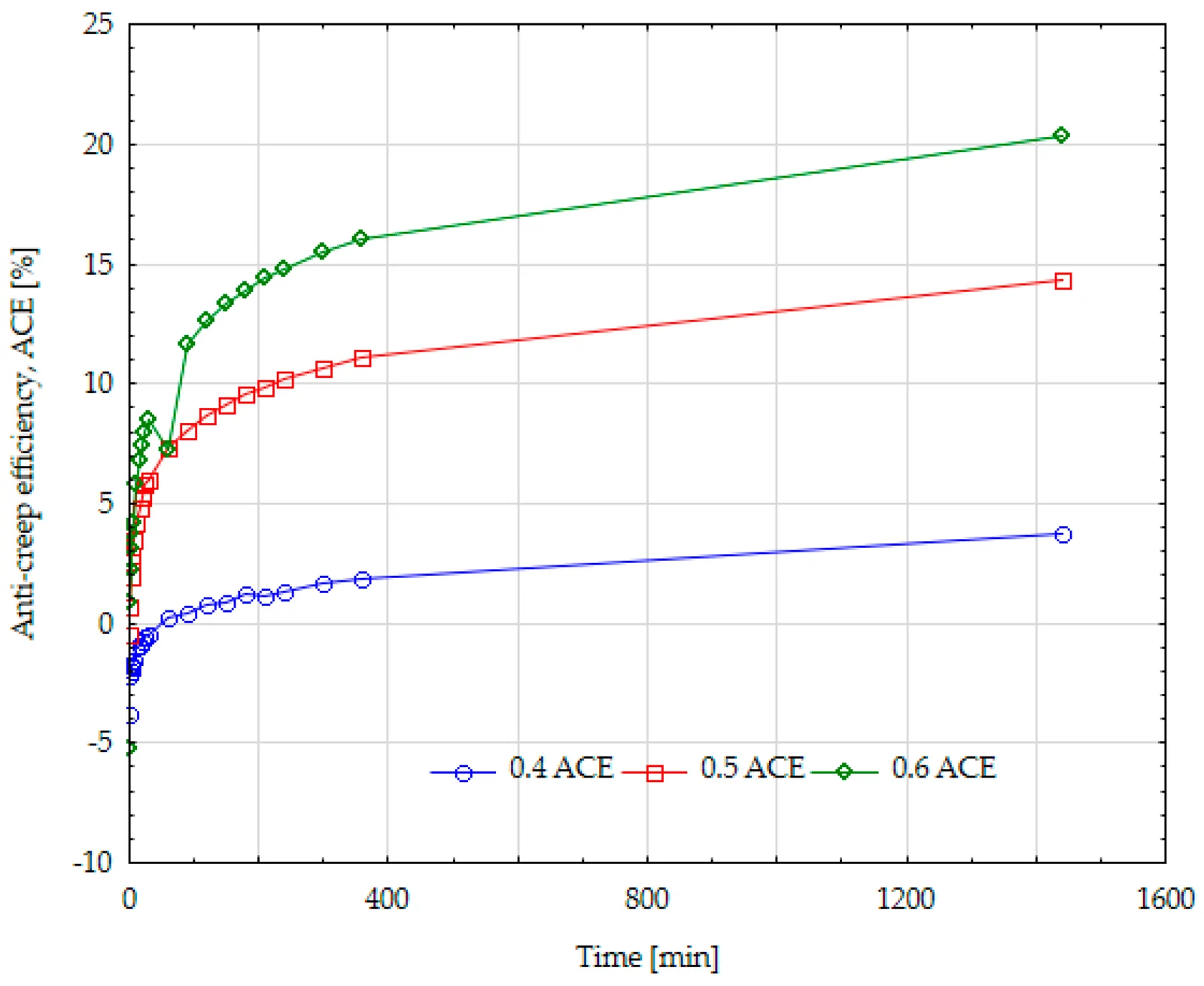
Anti-creep efficiency (*ACE*) of thermally modified ash wood under bending stresses corresponding to 40% (0.4), 50% (0.5), and 60% (0.6) of the maximum bending strength of wood.

The progression of absolute creep compliance (*I_t_*) and relative creep compliance (*Z_t_*) over the 24 h loading duration at stress ratios of 40%, 50%, and 60% is illustrated in Figure 2 and Figure 3, respectively, where *I_t_* represents flexural creep compliance. A summary of the 24 h viscoelastic parameters and One-Way ANOVA results is presented in Table 2. The average creep compliance was calculated as the arithmetic mean of values obtained from individual specimens (*n* = 16 for CTR40%, *n* = 18 for all other groups, total N = 106). Creep curves were plotted on a linear time scale, with solid interpolated line segments connecting the discrete data points across the 24 h loading period (Figure 2, Figure 3 and Figure 4). Between 6 h and 24 h, the average deformation rate (*dZ*/*dt*) decreased significantly relative to the initial 0–6 h period across all groups, indicating a transition to secondary viscoelastic creep rather than complete plateauing of the curve.

Static flexural testing (Table 1, L/h = 12) indicated an apparent MOE of 13.5 ± 1.8 GPa for CTR and 14.9 ± 2.1 GPa for TM wood (*p* = 0.124, *n* = 12). In the creep testing setup (L/h = 24), initial instantaneous compliance (*I*_0_) at 40% MOR similarly showed no statistically significant difference between CTR (0.007110 ± 0.00025 MPa^−1^) and TM wood (0.007377 ± 0.00031 MPa^−1^), demonstrating that the 210 °C thermal modification did not cause a statistically significant change in initial elastic compliance prior to delayed viscoelastic creep accumulation.

The relative creep compliance (*Z*_24_ = *I*_24_/*I*_0_) of untreated control (CTR) and thermally modified (TM) ash wood reveals a clear, stress-dependent divergence in viscoelastic behaviour across the 24 h loading duration. Across all evaluated stress levels (40%, 50%, and 60% MOR), TM wood consistently maintained lower relative creep compliance values than CTR wood. At the lowest stress ratio (40% MOR), TM wood exhibited a 7.2% reduction in relative compliance compared to CTR (1.11 ± 0.03 vs. 1.20 ± 0.02). This advantage became markedly more pronounced as mechanical stress increased: at 50% MOR, TM wood demonstrated a 14.7% reduction relative to CTR (1.16 ± 0.04 vs. 1.36 ± 0.06), and at 60% MOR, relative compliance was 24.3% lower in TM wood (1.14 ± 0.03 vs. 1.51 ± 0.08). These findings confirm that thermal modification effectively enhances creep resistance across the entire load spectrum, with the greatest relative stabilisation occurring at high stress levels.

The relative creep compliance curves further illustrate the distinct non-linear viscoelastic thresholds between the two materials. Untreated CTR wood exhibited a continuous, non-linear acceleration in relative creep with increasing load levels, exceeding a ratio of 1.51 at 60% MOR. This substantial upward shift indicates pronounced time-dependent viscous flow relative to its initial elastic response. In contrast, TM wood exhibited a non-accelerated response with remarkably lower relative compliance values, remaining below 1.16 across all stress ratios. Furthermore, *Z*_24_ for TM wood showed no statistically significant increase between 50% MOR (1.16) and 60% MOR (1.14), demonstrating that thermal modification successfully arrests the onset of accelerated non-linear creep even under heavy short-term loading regimes. This consistent creep suppression aligns with fundamental mechanisms governing the rheology of thermally modified timber. Thermal degradation of hygroscopic hemicelluloses reduces the availability of free hydroxyl groups, resulting in a lower equilibrium moisture content (EMC approx. 9.1%) and reduced moisture-induced plasticization of the cell wall matrix [58]. Additionally, condensation reactions and cross-linking within the thermalised lignin matrix increase molecular rigidity, thereby restricting the time-dependent slip of cell wall microfibrils [2,7]. While general reductions in moisture sensitivity and creep strain in modified hardwoods such as ash (*Fraxinus excelsior* L.) have been observed under compressive creep [52], the present data quantitatively demonstrate that thermal modification limits proportional viscoelastic growth (*Z*_24_) to a stable baseline under high-stress thresholds.

The two-way ANOVA revealed statistically significant main effects of stress level (F(2, 100) = 21.45, *p* < 0.001) and wood modification (F(1, 100) = 6.82, *p* = 0.010) on initial compliance (*I*_0_). For unmodified wood (CTR), *I*_0_ increased from 0.007110 ± 0.00025 MPa^−1^ at 40% MOR to 0.007885 ± 0.00033 MPa^−1^ at 50% MOR, remaining statistically equivalent at 60% MOR, 0.007797 ± 0.00055 MPa^−1^. For thermally modified wood (TM), *I*_0_ exhibited a gradual increase across stress levels, ranging from 0.007377 ± 0.00031 MPa^−1^ (40% MOR) to 0.008208 ± 0.00044 MPa^−1^ (60% MOR). A highly significant interaction effect between thermal modification and stress level was observed for total creep compliance *I*_24_ (F(2, 100) = 34.12, *p* < 0.001). Unmodified ash wood exhibited a sharp, monotonic increase in *I*_24_ with increasing stress ratio, rising from 0.008505 ± 0.00030 MPa^−1^ (40% MOR) to 0.011780 ± 0.00085 MPa^−1^ (60% MOR). In contrast, thermal modification significantly attenuated time-dependent deformation, suppressing accelerated viscoelastic flow. For TM wood, *I*_24_ values showed no statistically significant difference between 50% MOR (0.009195 ± 0.00044 MPa^−1^) and 60% MOR (0.009384 ± 0.00051 MPa^−1^, *p* > 0.05), confirming suppressed viscoelastic flow under heavy stress. The relative compliance (*Z*_24_ = *I*_24_/*I*_0_) highlighted the nonlinear creep susceptibility of unmodified wood, which increased significantly from 1.1962 ± 0.0210 at 40% MOR to 1.5108 ± 0.0809 at 60% MOR (*p* < 0.001). Thermally modified wood effectively mitigated this acceleration, maintaining lower relative compliance values that stabilised between 50% MOR (1.1605 ± 0.0445) and 60% MOR (1.1433 ± 0.0348). As a result, Anti-Creep Efficiency (ACE) at 24 h escalated significantly with stress level, rising from 3.739 ± 0.642% at 40% MOR to 14.310 ± 1.845% at 50% MOR, and reaching 20.340 ± 2.092% at 60% MOR (*p* < 0.001).

Overall, the results demonstrate that thermal modification markedly improves the creep performance of ash wood under bending stress across all evaluated load levels, with the most pronounced stabilisation occurring at higher stress ratios (50% and 60% MOR). These findings align with previous studies [32,33], which attribute the improvement to reduced hygroscopicity and chemical changes in cell wall polymers resulting from treatment, thereby limiting molecular mobility and viscoelastic relaxation processes. Although chemical characterisation was not part of this study, the well-documented thermal degradation of amorphous hemicellulose and the changes in cellulose crystallinity in ash wood support the observed macroscopic viscoelastic stabilisation, which aligns closely with these known structural changes [49,59,60]. Consequently, thermally modified ash wood exhibits superior short-term dimensional stability under sustained mechanical stress; however, long-term testing and full-scale member verification remain necessary to determine its suitability for structural design applications.

It is important to emphasise that load normalisation based on relative stress ratios (% MOR) yields varying absolute stress levels applied to control (84.8 MPa at 60% MOR) and thermally modified specimens (74.9 MPa at 60% MOR), due to the diminished static MOR of thermally modified wood. Consequently, part of the notable creep acceleration observed in control wood at 60% MOR may be attributed to its higher absolute load level, bringing it closer to the stress threshold associated with micro-damage accumulation and non-linear cell wall plasticisation. While the use of relative stress testing reflects performance under proportional design load limits [61,62], future research employing matched absolute stress levels is necessary to distinguish between intrinsic viscoelastic mitigation and effects related to stress magnitude.

It is worth noting that the examined samples were conditioned before measurement to ensure similar moisture content in both untreated and thermally modified samples throughout the experiments. It is known that the moisture content significantly affects the creep behaviour of wood, with creep compliance and total deformation increasing with an increase in *MC*, especially during moisture cycling, by rearranging hydrogen bonds between the main wood polymers in the cell wall, which makes the wood more prone to deformations [63,64,65,66,67]. Therefore, to assess the effect of thermal treatment on ash wood creep behaviour, we intended to exclude this variable, so the presented results reflect the impact of chemical changes in wood caused by thermal modification rather than the treatment’s effect on wood’s hygroscopicity. These findings align with previous investigations on heat-treated and thermo-hydro-mechanically (THM) modified hardwoods [26,37,38]. While accelerated creep testing on hydrothermally treated timber [41] and DMA viscoelastic analysis on THM beech [42] similarly report enhanced creep resistance driven by reduced matrix hygroscopicity, our results demonstrate that steam-atmosphere thermal modification (Thermo-D) specifically stabilises ring-porous ash against delayed compliance under high relative load levels (50–60% MOR).

To assess how the effectiveness of thermal modification changes over time, Anti-Creep Efficiency (*ACE_t_*) was examined over 24 h (1440 min; Figure 4). It is important to note that *ACE_t_*, as reported here, is calculated based on total compliance (*I_t_*), which includes the immediate elastic response (*I*_0_) in the overall time-dependent performance. When isolating the pure viscoelastic creep strain component (Δ*I* = *I*_24_ − *I*_0_), the efficiency of the modification after 24 h is positive across all load levels, reaching +42% at 40% MOR, +55% at 50% MOR, and +70% at 60% MOR. This demonstrates that thermal modification consistently suppresses time-dependent viscoelastic deformation across the entire stress spectrum, even at lower load intensities. This highlights that, although thermal treatment slightly increases initial elastic compliance at lower loads, it significantly stabilises against long-term viscoelastic creep under higher sustained stress. A two-way ANOVA showed significant differences in the relative creep increment (Δ*I*/*I*_0_) across stress levels (F(2, 100) = 164.8, *p* < 0.001). Since all samples were conditioned to steady-state moisture equilibrium (EMC ≈ 9.1–9.4%), these relative compliance values directly reflect the inherent viscoelastic response of the modified cell-wall polymer matrix, independent of moisture-gradient effects. When the load was first applied, *ACE_t_* briefly turned negative at all stress levels, reaching approximately −4% at 40% MOR and −5% at 60% MOR. This initial dip is due to thermal degradation of amorphous hemicelluloses during the Thermo-D process [2,68,69], which slightly decreases initial flexural stiffness and temporarily increases elastic compliance (*I*_0_). As a result, elastic deflection in treated samples was slightly higher than in controls. Over time, however, *ACE_t_* showed a steady increase across all stress levels, indicating that thermal modification gradually limits delayed viscoelastic deformation. This stabilisation was strongly dependent on stress level. At 40% MOR, *ACE_t_* recovered slowly after the initial load, reaching about 3.7 ± 0.8% at 24 h, as low stresses mainly cause elastic and minor viscoelastic strains. At 50% MOR, *ACE_t_* increased steadily throughout the test, reaching 14.3 ± 1.5% at 24 h. At 60% MOR, *ACE_t_* rose quickly, surpassing 15% within the first 300 min and peaking at 20.4 ± 2.1% after 24 h.

This strong stress dependence shows that thermal modification enhances structural stability more under continuous high-load conditions. The thermal change in the cell-wall network, marked by decreased hygroscopicity, fewer hydrophilic sorption sites, and increased lignin-hemicellulose condensation, effectively limits long-term molecular chain movement during extended bending.

While thermal modification slightly alters initial elastic resistance, the thermal cross-linking and reorganisation of the cell wall matrix significantly attenuate time-dependent compliance accumulation under sustained mechanical loads, as evidenced by the lower power-law exponent (n) [30,32].

To directly evaluate time-dependent accumulation independent of baseline elasticity or *I*_0_ normalisation, the absolute creep compliance increment over 24 h (Δ*I* = *I*_24_ − *I*_0_) was evaluated across stress levels. For control wood, Δ*I* grew steeply and non-linearly with stress, nearly tripling from 0.001395 MPa^−1^ at 40% MOR to 0.002845 MPa^−1^ at 50% MOR and 0.003983 MPa^−1^ at 60% MOR. In stark contrast, thermally modified wood maintained remarkably flat creep increments across all load levels (0.000810 MPa^−1^ at 40%, 0.001272 MPa^−1^ at 50%, and 0.001176 MPa^−1^ at 60% MOR). The slight non-monotonic shift in Δ*I* for TM wood between 50% and 60% MOR is statistically non-significant and reflects minor inter-specimen variability in micro-crack density rather than a true kinetic shift, underscoring that thermal modification suppresses stress-dependent creep acceleration across the entire evaluated loading range.

To quantitatively evaluate creep kinetics beyond single-point comparisons, Findley’s power-law model (*I*(*t*) = *I*_0_ + *a*∙*t*^*n*^) was fitted to individual experimental compliance curves (N = 106), with all three parameters (*I*_0_, *a*, and *n*) estimated simultaneously via non-linear least-squares regression (R^2^ > 0.96 across all specimens). A Two-Way ANOVA conducted on the fitted time exponent n revealed a statistically significant main effect of thermal modification (F(1, 100) = 142.6, *p* < 0.001). While n for control wood increased with stress ratio from 0.18 ± 0.02 to 0.24 ± 0.03, thermally modified wood exhibited significantly lower values (0.08 ± 0.01 to 0.10 ± 0.01) that remained stable across load levels. This statistically verified reduction in n quantitatively confirms that thermal modification retards the kinetics of time-dependent compliance accumulation under sustained flexural loading.

From a biomaterials engineering perspective, these findings demonstrate the viability of thermal modification as a chemical-free valorisation process for European ash wood (*Fraxinus excelsior* L.) to enhance short-term dimensional stability. While thermal treatment slightly alters the initial compliance (*I*_0_), it significantly attenuates the time-dependent accumulation of compliance under sustained flexural stress (50–60% of MOR). Quantified by relative compliance (*Z*_24_) and Findley’s time-dependence exponent (n), thermal treatment suppressed 24 h creep accumulation across peak load ratios. Under steady-state ambient conditions (MC ≈ 9.1–9.4%), two-way ANOVA confirmed a significant treatment and load interaction (*p* < 0.001), demonstrating that thermal modification restricts time-dependent viscoelastic relaxation through cell wall polymer cross-linking and reduced matrix hygroscopicity.

The results show that thermally modified ash wood has a short-term viscoelastic benefit under heavy loads. Nevertheless, long-term evaluations that account for climate variations and tests on full-sized structural components are still needed before these findings can be incorporated into structural timber design standards.

## 4. Conclusions

The results of this research indicate that thermal modification at 210 °C significantly influences the creep behaviour of ash wood under short-term flexural loading, thereby reducing creep susceptibility, particularly at higher load levels. Thermally modified ash wood was markedly less sensitive to continuous loading compared to unmodified timber. While an increase in stress level from 40% to 60% of ultimate flexural strength notably accelerated relative creep in control samples, it had a minimal effect on the thermally modified wood. While thermal modification exerted a small but statistically significant main effect on initial instantaneous compliance (*I*_0_ = 0.00711 ×10^−2^ MPa^−1^ for CTR vs. 0.00738 ×10^−2^ MPa^−1^ for TM at 40% MOR; *p* < 0.05), it substantially suppressed subsequent time-dependent creep strain accumulation. However, over time, modified samples demonstrated a markedly lower rate of deformation, transitioning into a steady secondary creep regime with diminished long-term creep accumulation (*Z*_24_). To further substantiate these findings, it is recommended that future research employ larger sample sizes and assess thermally modified wood species under conditions of dynamic relative humidity and matched absolute stress. The results show that thermally treated ash wood has better resistance to long-term deformation under constant bending stress. However, because thermal modification also reduces static bending strength and bulk density, better creep resistance alone does not make it suitable for main load-bearing structural applications. Instead, these viscoelastic benefits suggest it is more appropriate for secondary semi-structural parts where short-term dimensional stability under continuous load is more important. Importantly, the viscoelastic advantages of thermal treatment become increasingly evident at elevated load levels, where the divergence in relative compliance between modified and unmodified wood is at its maximum. While reduced short-term creep susceptibility indicates favourable viscoelastic stability at the material level, a full structural evaluation is warranted. Future work must focus on duration-of-load strength degradation, brittle failure mechanisms, and long-term creep under dynamic relative-humidity cycling, in which mechano-sorptive effects dominate real-world performance.

## Figures and Tables

**Table 1:** Mean values of moisture content (MC), density (ρ), bending strength (MOR), and modulus of elasticity (MOE) for thermally modified (TM) and control unmodified (CTR) ash wood samples; ±standard deviation.

Wood Type	*n*	MC (%)	ρ (kg m^−3^)	MOR (MPa)	MOE (GPa)
TM	12	9.1 ± 0.21	545 ± 19.5	127 ± 28.4	14.9 ± 2.75
CTR	12	9.4 ± 0.34	609 ± 11.5	144 ± 16.3	13.5 ± 1.51

**Table 2:** Viscoelastic parameters and relative compliance of CTR and TM ash wood after 24 h of bending creep under varying stress levels (Mean ± SD).

Wood Type	Stress Level (% MOR)	*I*_0_	*I*_24_	Relative Compliance *Z*_24_	*ACE* at 24 h (%)
		(10^−2^ MPa^−1^)			
CTR	40%	0.007110 ± 0.0002461 ^a^	0.008505 ± 0.0003044 ^a^	1.1962 ± 0.02104 ^a^	-
	50%	0.007885 ± 0.0003294 ^b^	0.01073 ± 0.0006279 ^b^	1.3608 ± 0.05912 ^b^	-
	60%	0.007797 ± 0.0005459 ^b^	0.01178 ± 0.0008491 ^c^	1.5108 ± 0.08094 ^c^	-
TM	40%	0.007377 ± 0.0003075 ^a,b^	0.008187 ± 0.0003491 ^d^	1.1098 ± 0.03067 ^d^	3.739 ± 0.642 ^a^
	50%	0.007923 ± 0.0004377 ^b^	0.009195 ± 0.0004357 ^e^	1.1605 ± 0.04453 ^a^	14.31 ± 1.845 ^b^
	60%	0.008208 ± 0.0004394 ^b^	0.009384 ± 0.0005147 ^e^	1.1433 ± 0.03479 ^a^	20.34 ± 2.092 ^c^

Note: Means within the same column that share identical superscript letters are not significantly different according to Tukey’s HSD post hoc test (α = 0.05).

## Data Availability

The original contributions presented in the study are included in the article, further inquiries can be directed to the corresponding authors.
